# Atypical Presentation of Congenital Triangular Alopecia: A Case Series in Italy

**DOI:** 10.5826/dpc.1004a122

**Published:** 2020-10-26

**Authors:** Michela Starace, Miriam Anna Carpanese, Diego Abbenante, Francesca Bruni, Bianca Maria Piraccini, Aurora Alessandrini

**Affiliations:** 1 Department of Experimental, Diagnostic and Specialty Medicine, Division of Dermatology, University of Bologna, Italy

**Keywords:** congenital triangular alopecia, scalp, trichoscopy, atypical

## Abstract

**Background:**

Congenital triangular alopecia (CTA) is a benign, asymptomatic, nonprogressive, localized and noncicatricial type of alopecia that is usually first noted during infancy or childhood. The pattern of hair loss is traditionally described as triangular, oval or lancet shaped with apex toward the vertex.

**Objective:**

We present a case series of CTA located in unusual sites.

**Patients and Methods:**

We performed trichoscopy in 78 patients with CTA. From this group, we selected 10 individuals (4 males and 6 females) whose disease was not localized on the typical scalp area.

**Results:**

The alopecic area was located on the occipital region in 5 patients, the parietal region in 4 patients, at the vertex in 1. With trichoscopy, vellus hairs were detected in all patients, and evidence of empty follicles was noticed only in 3 patients.

**Conclusions:**

In contrast with the preconceived notion that all CTAs are frontotemporal, our case series points out that this disease could be localized in other scalp sites

## Introduction

Congenital triangular alopecia (CTA) is a benign, asymptomatic, nonprogressive, localized and noncicatricial type of alopecia that is usually first noted during infancy or childhood. Previously other names have been used to describe this condition, such as Brauer nevus or temporal triangular alopecia. CTA most commonly affects the frontotemporal region of the scalp in a unilateral pattern, more frequently involving the left side. In a minority of cases a bilateral involvement has been reported [1]. The pattern of hair loss is traditionally described as triangular, oval, or lancet-shaped with the apex toward the vertex [2]. The diagnosis is mainly based on its clinical appearance, and usually pathology is not needed to confirm the diagnosis. Trichoscopy can be useful in excluding other diseases characterized by localized alopecia [3], such as alopecia areata. Herein, we present a case series of CTA located in unusual sites.

## Methods

During the period from 2012 to 2020, at outpatient hair consultation of the Department of Experimental, Diagnostic and Specialty Medicine at the University of Bologna, we diagnosed 78 patients with CTA. In all patients we performed trichoscopy using a FotoFinder dermatoscope. From this group, we selected 10 individuals (12.8%) whose disease was not localized in the typical scalp area. All the patient’s data are summarized in Table 1.

## Results

**Figure 1:**
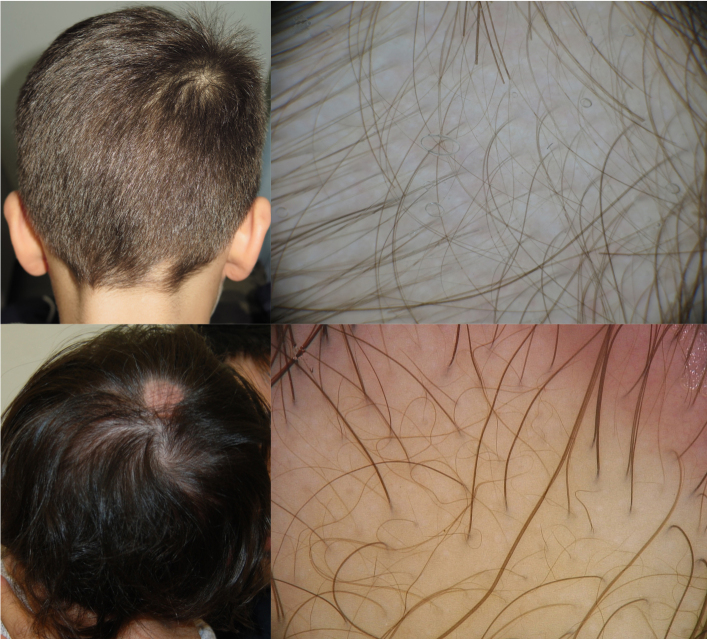
Two cases of our series: (A) occipital CTA; (B) vertex CTA. With trichoscopy, vellus hairs are evident (C, D), but also empty follicles (D). CTA = congenital triangular alopecia.

In this group there were 6 males and 4 females with a mean age of 3.5 years. Regarding the location on the site, the alopecic area was located on the occipital region in 5 patients, the parietal region in 4 patients, and at the vertex in 1 patient (Figure 1, A and B). With trichoscopy, vellus hairs were detected in all patients, and evidence of empty follicles was noticed only in 3 patients (Figure 1, C and D).

## Discussion

CTA is a non-scarring type of alopecia. Its exact incidence is not known, but some studies reported an estimated rate of 0.11% [4]. Most CTAs present in children between 3 and 6 years old, but it can also occur in adult patients. According to Kumar Dey et al [5], as of 2016, only 127 cases had been reported worldwide. It usually appears in a sporadic form, but familial cases have been described [6,7]. In a smaller percentage of cases, about 15%, CTA can be associated with other disorders or syndromes, such as phacomatosis pigmentovascularis [8,9]. To date, the etiology is unknown, and no specific treatments are available for this benign condition. Despite the typical presentation of CTA as a triangular or oval patch of circumscribed alopecia localized in the frontotemporal area, some authors reported atypical locations of CTA in the mid-frontal region of the scalp [10], the occipital area [11], the left temporo-parietal-vertex region of the scalp [12], and the eyebrows [13]. The diagnosis of CTA is mainly based on its clinical appearance and location. Pathology shows miniaturized follicles that replace terminal hairs, with an increased proportion of vellus or indeterminate hairs. The total number of follicle units is in the normal range [1], but it is not usually performed. The differential diagnoses of the disease include alopecia areata, trichotillomania, traction alopecia, and congenital aplasia cutis.

Several cases reported in literature show that CTA could be misdiagnosed and incorrectly treated as other forms of focal alopecia, principally alopecia areata [11,14]. For this reason, trichoscopy can be useful, avoiding scalp biopsy or useless treatments. Typical trichoscopic findings include normal follicular openings with vellus hairs covering the alopecic area and terminal hairs on the outskirt of the lesion [15]. Classic signs of alopecia areata, black or yellow dots or exclamation marks, are absent in CTA. Inui et al [16] proposed 4 diagnostic criteria for CTA: triangular or spearshaped area of alopecia involving the frontotemporal region of the scalp; trichoscopic features of normal follicular openings with vellus hair surrounded by normal terminal hair and absence of yellow and black spots, dystrophic hairs and decreased follicular openings; and persistence of no significant hair growth after dermoscopic and clinical confirmation of the existence of vellus hairs. Our cases fulfill all the criteria for CTA except the site of involvement.

## Conclusions

In contrast with the preconceived notion that all CTAs are frontotemporal, our case series points out that this disease could be localized in other scalp sites. This focus is important because CTA is an underdiagnosed condition, and in order to avoid redundant therapies, it should be included in differential diagnosis when evaluating a circumscribed hairless patch on the scalp.

## Figures and Tables

**Table 1:** Clinical Data from Patients With CTA Localized on Atypical Scalp Area

Case Number	Age	Sex	Site	VDS
1	3	M	Occipital	Vellus hair
2	5	M	Parietal	Vellus hair
3	1	F	Occipital	Vellus hair and empty follicles
4	4	F	Parietal	Vellus hair
5	5	F	Occipital	Vellus hair
6	4	M	Occipital	Vellus hair and empty follicles
7	2	F	Parietal	Vellus hair and empty follicles
8	3	M	Parietal	Vellus hair
9	4	M	Occipital	Vellus hair
10	3	F	Vertex	Vellus hair

CTA = congenital triangular alopecia; VDS = videodermoscopy
